# The Parasites of Man

**Published:** 1886-12

**Authors:** 


*The Parasites of Man.* By RUDOLF LEUCKART. Translated by W. E. HOYLE, M.A., M.R.C.S. Pp. 771. Edinburgh: YOUNG J. PENTLAND. 1886.

We are equally indebted to translator and to publisher for placing this splendid, almost monumental work in the hands of English readers. The English literature of human parasites is represented almost entirely by the translation of Küchenmeister's work, now almost obsolete, and by the various treatises of Cobbold, which are by no means exhaustive. The work before us not only fills a gap in our literature, but gives us an opportunity of exhausting available knowledge, and of studying the original work of a master.

For thirty years the author has laboured at his subject. Many hundreds of animals have been used for his helminthological experiments, and the number of parasites investigated was much greater. The work everywhere bears the impress of laborious, original, and accurate observation, with the natural concomitants of clear language, vigorous description, and scientific classification.

The book, though devoted mainly to the entozoa infesting man, offers also an almost complete survey over the present state of the zoology of parasites. The first part of the work gives a general account of the natural history of parasitic life; then follows a systematic account of the parasites infesting man, prefixed in each case by a general sketch of the structure and life history of the groups to which they belong. This volume deals with protozoa and cestoda; a second volume, now being revised by the author for a second German edition, will be translated from proof-sheets by Mr. Hoyle, and published simultaneously in England and in Germany.

No student of helminthology can afford to be without Leuckart's work.

